# Supplementary material for: Factors associated with anaemia in pregnancy: A retrospective cross-sectional study in the Bolgatanga Municipality, northern Ghana
Source: PLoS One. 2023 May 25;18(5):e0286186. doi: 10.1371/journal.pone.0286186 (PMC10212080; doi:10.1371/journal.pone.0286186)
Supplement: Attachment — Submitted filename: Responses to Reviewers (1).docx [file pone.0286186.s001.docx]

RESPONSE TO EDITOR’S AND REVIEWER’S COMMENTS

| **No.** | **Reviewer’s Comments** | Response to Comment |
| --- | --- | --- |
| **Editor’s instructions** | | |
| 1 | Please ensure that your manuscript meets PLOS ONE's style requirements, including those for file naming. | We have reformatted the manuscript to ensure that it meets PLOS ONE’s style requirements |
| 2 | You indicated that you had ethical approval for your study. In your Methods section, please ensure you have also stated whether you obtained consent from parents or guardians of the minors included in the study or whether the research ethics committee or IRB specifically waived the need for their consent. | We thank the editor for this comment. Five respondents were aged between 16 and 17 of which four were married. In the study area, those who are married often live with their husbands and are often not treated as minors. Since they are married, their parents no longer take decisions for them and it is often difficult to reach to their parents for consent. We, therefore, obtained informed consent from the respondents themselves and/or their non-minor husbands (acting as guardians). For the one who was not married, we obtained informed consent from her and her parents. We have therefore revised that portion of the ethics statement to read  “*Written informed consent was obtained from all respondents and/ their parents/guardians after they were given information on the purpose of the study and what was required of them as study participants*” (See lines 187 - 189.) |
| 3 | Your ethics statement should only appear in the Methods section of your manuscript. If your ethics statement is written in any section besides the Methods, please move it to the Methods section and delete it from any other section. Please ensure that your ethics statement is included in your manuscript, as the ethics statement entered into the online submission form will not be published alongside your manuscript. | The ethic statement has been moved to the methods section. The ethics statement now appears only in the methods section. This shown on lines 184 - 189 |
| 4 | Please include captions for your Supporting Information files at the end of your manuscript, and update any in-text citations to match accordingly. Please see our Supporting Information guidelines for more information: <http://journals.plos.org/plosone/s/supporting-information>. | We no longer have supporting information files in the manuscript. We have updated the intext citations |
| **Reviewer’s Comments** | | |
| 1 | Line 1 (Title): The Bolgatanga Municipality is a small part of what we know as northern Ghana, which includes 5 regions, namely the Northern, Savannah, North-East, Upper East, and Upper West regions. Thus, authors should insert "Bolgatanga Municipality" in the title, so it reads: "Factors associated with anaemia in pregnancy: A retrospective cross sectional study in the Bolgatanga Municipality in northern Ghana". | We are very grateful to the reviewer for this observation. We have now included Bolgatanga Municipality in the title of the manuscript as recommended by the reviewer. The title of the manuscript now reads “*Factors associated with anaemia in pregnancy: A retrospective cross-sectional study in the Bolgatanga Municipality in northern Ghana***”** as shown in lines 1 - 2. |
| 2 | Line 35: It would be helpful to include the mean (or median) gestational weight at registration. | The authors agree with the reviewer that gestation weight at registration is an important and plausible determinant of AIP. Unfortunately, we did not extract data on gestational weight during the data collection. We, therefore, apologize for this omission. We have included this as a limitation.  See Line 412-414: “*Data on other important variables such as gestational weight at ANC registration were not gathered and hence such variables were excluded in the analysis*” |
| 3 | Line 36-38: Authors' use of "after haemodilution" is confusing. Why not use something like "after WHO adjustment" or simply "using anemia cut off of 11.0 g/dl" versus "using anemia cut-off of 10.5 g/dl"? | We thank the reviewer for the observation. In fact, what we mean is after adjusting for Haemodilution but not “after Haemodilution” as erroneously captured. We have therefore revised the relevant parts of the manuscript to read “after the WHO recommended adjustment for Haemodilution” or simply “after adjusting for Haemodilution” as seen in line 38 – 40 and other sections |
| 4 | Line 39-40: Authors should use was/were "associated with increased odds" or "associated with decreased odds" ..... | We are grateful to the reviewer for this recommendation in both the abstract and in the results section of the manuscript. See line 40 - 52. |
| 5 | Line 90-92: Readers do not believe that "only two studies done elsewhere" (Refs 46 and 47) have ever reported the hemoglobin concentration and anemia status of women at more than one time point during pregnancy? | We have replaced “only two studies done elsewhere” with “only few studies conducted elsewhere”. In our literature search, we identified some studies that assessed AIP at one point of gestation and two studies assessing AIP at two points of gestation but to the best of our knowledge we did not identify any study that assessed AIP using the three WHO recommended points for haemoglobin monitoring during antenatal care. See lines 98 - 102 |
| 6 | Line 92-94: This (in addition to Line 90-92) is a poor description of study rationale and appears to suggests that authors are not familiar with the Hb/anemia literature of pregnant women in Ghana. I can give you at least one of such studies in Ghana: Adu-Afarwuah S, Lartey A, Okronipa H et al. (2017) Impact of small-quantity lipid-based nutrient supplement on hemoglobin, iron status and biomarkers of inflammation in pregnant Ghanaian women. Maternal & Child Nutrition (2017), 13, e12262.. Was the primary aim of the study not to determine the factors associated with anemia at first antenatal booking, 28 weeks of pregnancy, and 36 weeks of pregnancy in the Bolgatanga Municipality? | It is true that the primary aim of our study was to determine the factors associated with anaemia at first antenatal booking, 28 weeks of pregnancy, and 36 weeks of pregnancy in the Bolgatanga Municipality as stated in the last paragraph of the introduction section. We have made significant revisions to the rationale for study and have included references to the study indicated by the reviewer. We, however, wish to stress that the gap we tried pointing out is the assessment of haemoglobin at all the three points of gestations but not just at more than one point of gestation.  *“However, only few studies conducted elsewhere [46,47] assessed changes in haemoglobin levels and anaemia status of women in the course of pregnancy. In Ghana, Adu-Afarwuah et al, assessed the impact of iron supplementation on haemoglobin levels of pregnant women using a randomised controlled trial involving haemoglobin checks at two points in gestation. We could not identify studies in Ghana assessing* *haemoglobin levels and anaemia status among pregnant women at all the three WHO recommended points for haemoglobin check during ANC and their determinants in the course of gestation. Some studies assessed AIP at one point of gestation [48,49] and others at two points [50,51], but no study assessed the phenomenon at all the three recommended haemoglobin check points during ANC. These three points are used for decision making, hence, knowledge on anaemia prevalence and its determinants at the three stages of pregnancy would be useful in the care process. This study, therefore, assessed haemoglobin levels and the prevalence of anaemia at ANC registration, at 28 weeks and 36 weeks of gestation and the socio-economic factors associated with AIP at these stages of pregnancy”*  See lines 98 - 111 |
| 7 | Line 124-126: In line 110, "haemoglobin checked at registration was only about 52% at 36 weeks of gestation". So, given this "retrospective cross-sectional study", did authors randomly select participants from among those with Hb values at all 3 time points (first book, 28 wk, and 36 wk)? | Having Hb values at all three time points was not a criterion for sampling. We clearly stated in the methods section that “*All women in 2020 who registered for ANC before 28 weeks and had reached 36 weeks of gestation during the period of data collection were eligible to be included in the study*” (line 128 - 130).  We now inserted the following statement to clarify the sampling ‘’all participants were randomly sampled at or after 36 weeks of gestation” (line 131 - 132)  In addition to this, we already included the following statement which further clarifies our sampling “*A proportionate sample, based on the number of ANC registrants per facility was drawn from each of the 28 facilities offering ANC in the Municipality. At the facility level, simple random sampling using random numbers was used to select the number of respondents allotted to each facility. The ANC registers of the various facilities constituted the sampling frame (lines* 139 - 141) |
| 8 | Line 132-134: This is a "retrospective" study, so this part is confusing, i.e., "a structured questionnaire was used to collect data on household food security and additional sociodemographic characteristics including household assets, that were not captured in the medical records." Authors should describe clearly how the women were contacted for the survey. Were the hospital records reviewed before or after the survey? For a "retrospective cross-sectional study", were all participants enrolled after 36 weeks of pregnancy? | As already indicated in response to the previous comment, all participants were enrolled at or after 36 weeks of gestation. This has already been captured in the manuscript. Both the records review and questionnaire administration were done at one point in time. The following information in Line 157-165 address this:  *“The researchers first made contact visits to the health facilities to identify eligible respondents who were then sampled for the study. The midwifes at the sampled health facilities, who know the schedules of the women for ANC, assisted the data collectors in the final contact and selection of the respondents. We first extracted the relevant data from the ANC records of each participant and immediately administered the questionnaire to the participant. The records reviews and questionnaire administration were, therefore, done at the same point in time. As a retrospective study, the records review and the questionnaire gathered data on issues within the course of the pregnancy”* |
| 9 | Line 142-143: At what time point (in terms of gestational age) were the women contacted? Were (some) women contacted before 36 wk of pregnancy? If so, how is this a "retrospective" study? | All women were contacted at or after 36 weeks of gestation. |
| 10 | Line 155-156: Comparison of means at 3 time points: it is confusing how they used a dependent (paired) t-test here. Please provide more details on how the analyses were organized. | We thank the reviewer for this comment. We have removed these results from the manuscript so that the study can directly focus only on its objectives of assessing AIP prevalence and its determinants. |
| 11 | Line 158-159: Comparison of percentages at 3 time points. Please provide details of how the analyses were organized, eg, (1 v 2, 1 v 3, and 2 v 3?. | We thank the reviewer for this comment. This comparison has also been removed. |
| 12 | Line 160-162: Please describe how the predictors were selected for the final binary regression models. | We wish to thank the reviewer for this comment. All predictors were included based on their relevance to AIP cause and prevention or association with it as reported by previous studies and based on researchers’ prior knowledge of the context of the study area and setting. This is indicated on lines 179-181 as “All predictors were included into the model based on their relevance to explaining AIP as reported by previous studies and their contextual appropriateness” |
| 13 | Table 3: What are the P-values comparing? | We have removed the P-values in Table 3. We agree with the reviewer than they are not needed. |
| 14 | Table 4 is very confusing: a. Why did authors decide to separate non-anemic versus anemic women? Was this part of the objective, in addition to examining mean Hb concentrations at the 3 time points? b. What are the "adjusted" and "unadjusted" Hb values? How was the adjustment done? I see the comparisons were: Registration vs 28 wk, Registration vs 36 wk, and 28 wk v 36 wk. I'm sure readers this presentation boring; the table is long and difficult to follow (with the use of "adjusted" and "unadjusted" Hb). Maybe should consider using a line graph to present these data. | We agree with the reviewer that the segregation of the analysis by anaemic and non-anaemic status in table 4 does not add much to addressing our research objectives. We have, therefore, deleted the information in the table and in the text relating to this analysis. |
| 15 | Table 5: How did authors select predictor variables for the logistic regression models? Did they simply throw all the background socioeconomic and food insecurity, etc., variables into the models? That is why this table is so big. | We have now indicated in the methods section that “*All predictors were included into the model based on their relevance to explaining AIP as reported by previous studies and their contextual appropriateness” (See line 179 - 181.)* |
